# Expanding Diversity of *Firmicutes* Single-Strand Annealing Proteins: A Putative Role of Bacteriophage-Host Arms Race

**DOI:** 10.3389/fmicb.2021.644622

**Published:** 2021-04-20

**Authors:** Kamil Steczkiewicz, Eric Prestel, Elena Bidnenko, Agnieszka K. Szczepankowska

**Affiliations:** ^1^Institute of Biochemistry and Biophysics PAS, Warsaw, Poland; ^2^Micalis Institute, INRAE, AgroParisTech, Université Paris-Saclay, Jouy-en-Josas, France

**Keywords:** single strand annealing proteins (SSAP), phage recombinase, *Firmicutes* bacteriophages, CLANS, CRISPR/cas, abortive intection, Sak3/DUF1071, phage-bacteria arms race

## Abstract

Bacteriophage-encoded single strand annealing proteins (SSAPs) are recombinases which can substitute the classical, bacterial RecA and manage the DNA metabolism at different steps of phage propagation. SSAPs have been shown to efficiently promote recombination between short and rather divergent DNA sequences and were exploited for *in vivo* genetic engineering mainly in Gram-negative bacteria. In opposition to the conserved and almost universal bacterial RecA protein, SSAPs display great sequence diversity. The importance for SSAPs in phage biology and phage-bacteria evolution is underlined by their role as key players in events of horizontal gene transfer (HGT). All of the above provoke a constant interest for the identification and study of new phage recombinase proteins *in vivo*, *in vitro* as well as *in silico*. Despite this, a huge body of putative *ssap* genes escapes conventional classification, as they are not properly annotated. In this work, we performed a wide-scale identification, classification and analysis of SSAPs encoded by the *Firmicutes* bacteria and their phages. By using sequence similarity network and gene context analyses, we created a new high quality dataset of phage-related SSAPs, substantially increasing the number of annotated SSAPs. We classified the identified SSAPs into seven distinct families, namely RecA, Gp2.5, RecT/Redβ, Erf, Rad52/22, Sak3, and Sak4, organized into three superfamilies. Analysis of the relationships between the revealed protein clusters led us to recognize Sak3-like proteins as a new distinct SSAP family. Our analysis showed an irregular phylogenetic distribution of *ssap* genes among different bacterial phyla and specific phages, which can be explained by the high rates of *ssap* HGT. We propose that the evolution of phage recombinases could be tightly linked to the dissemination of bacterial phage-resistance mechanisms (e.g., abortive infection and CRISPR/Cas systems) targeting *ssap* genes and be a part of the constant phage-bacteria arms race.

## Introduction

The great genetic diversity and abundance of bacteriophages (phages) on earth determine their profound impact on the bacterial world and the biosphere on the whole (Srinivasiah et al., 2008; Kristensen et al., 2010; Hatfull and Hendrix, 2011). Rapid bacteria-phage co-evolution was suggested in order to explain the structure and dynamics of different bacterial populations and the extensive horizontal gene transfer (HGT) between different bacterial and phage (prophage) genomes (Canchaya et al., 2003; Pal et al., 2007). Thus, phages are commonly considered as a major driving force in bacterial evolution in different natural environments, including the human or animal body (Gómez and Buckling, 2011; De Paepe et al., 2014b; De Sordi et al., 2019).

DNA recombination is a keystone of HGT; the almost universal RecA recombinase encoded by nearly all bacterial genomes sequenced so far plays a vital role in this process (Rocha et al., 2005). Phages rarely encode classic RecA proteins but rather used to own recombinases—DNA single strand annealing proteins (SSAPs), which are diverse in sequence and phyletic distribution. Phage SSAPs substitute for RecA and manage the DNA metabolism at different steps of phage infection (Kuzminov, 1999; Szczepañska, 2009). The importance of phage-encoded SSAPs for HGT is now well documented and cannot be underestimated (De Paepe et al., 2014a).

Among the characteristic features of phage SSAPs is their efficient pairing activity of short DNA sequences (40–50 bp) and the ability to promote recombination between divergent DNA sequences (Murphy et al., 2000; Court et al., 2002; Noirot et al., 2003; Martinsohn et al., 2008). Another distinguishing and intriguing trait of phage SSAPs is their great diversity, presenting a deep contrast to the conserved bacterial RecA protein.

Initially three evolutionary distinct SSAP superfamilies (RecT/Redβ, Erf and Rad52) have been identified by bioinformatic analyses (Iyer et al., 2002) and a fourth one (Sak4) by experimental studies (Bouchard and Moineau, 2004). Later it has been proposed that the majority of 465 SSAPs identified within the annotated phage genomes archived in the ACLAME database (version 0.3) of mobile genetic elements can be classified into one of these three superfamilies: Rad52 (incorporating RecT/Redβ and Erf superfamilies, and the Sak-like protein family), Rad51 and Gp2.5 (Leplae et al., 2010; Lopes et al., 2010). Six phage SSAP families, namely UvsX, Gp2.5, Sak, RecT/Redβ, Erf, and Sak4 have been identified within these superfamilies (Lopes et al., 2010). At present, the total amount of annotated SSAPs continues to increase, not only because of the rising number of available phage/prophage sequences, but also due to the growing performance of methods of sequence analysis. Yet, a huge body of putative *ssap* genes is not taken into account and escapes classifications as it is not annotated as phage-related. Thus, a comprehensive updated classification of SSAPs comprising the current data and determination of the evolutionary relationships between different SSAPs are required.

Special attention in this subject was exercised by the use of some *ssap* genes for recombineering, an efficient method of *in vivo* genetic engineering first developed in *Escherichia coli* (Datsenko and Wanner, 2000; Ellis et al., 2001; Court et al., 2002; Sawitzke et al., 2007) and adapted for other, mainly Gram-negative, bacterial species (Derbise et al., 2003; Ranallo et al., 2006; Sawitzke et al., 2007; Lesic and Rahme, 2008; van Kessel et al., 2008). Up to now, the use of SSAPs for recombineering is limited to proteins from the RecT/Redβ SSAP family which co-occur with their native exonuclease. It is noteworthy that although RecT/Redβ recombinases of different origin can function in *E. coli* to catalyze oligonucleotide recombination, yet some at albeit barely detectable thresholds, the double-strand DNA recombination activities with their exonuclease partners have been shown to be inefficient (Datta et al., 2008). Information concerning other SSAPs, especially those encoded by phages and prophages of Gram-positive bacteria, is in short supply. All above justify the constant interest for the identification and study of new phage recombinase proteins *in vivo*, *in vitro* as well as *in silico*.

Although the extreme diversity of phage SSAPs and their large phyletic distribution are now generally recognized and documented, a rational explanation for this has never been proposed. A number of studies have analyzed the role of gene flow in the adaptation of phages to their hosts (Paterson et al., 2010), but the mechanisms and the driving force of evolution as well as the diversity of phage genes have been investigated to a lesser extent. At the same time, there is a significant amount of experimental data supporting the role of phage resistance mechanisms in phage-bacteria co-evolution and phage diversity (Buckling and Rainey, 2002; Labrie and Moineau, 2007; Sorek et al., 2008; Enikeeva et al., 2010; Koskella and Brockhurst, 2014; Broniewski et al., 2020).

Bacteria have developed different effective anti-phage defense strategies: both generalized and highly specific (Sorek et al., 2008; Labrie et al., 2010; Makarova et al., 2011). Among these, Clustered Regulatory Interspaced Short Palindromic Repeats (CRISPR)/Cas system and phage Abortive infection (Abi) are known to target specific phage functions essential for intracellular phage development. The most elaborated adaptive defense CRISPR/Cas systems provide immunity against phages and invasive genetic elements. The defense mechanism is based on the incorporation of short DNA sequences (proto-spacers) from the infecting phage into the CRISPR locus. Subsequently, CRISPR transcripts are processed into small interfering RNAs that guide the bacterial protein complex to cleave matching foreign DNA. Spacer sequences homologous to various phage genes were identified in sequenced bacterial genomes (Sorek et al., 2008). Abis are characterized by the blockage of a crucial step of phage intracellular development associated with the premature onset of cell death (Chopin et al., 2005; Labrie et al., 2010). The *abi* genes have been identified in different bacterial phyla, even if the majority of them were characterized for Gram-positive bacteria *Lactococcus lactis* (Chopin et al., 2005; Labrie et al., 2010).

In the present study, we focused on the identification, classification and analysis of SSAPs encoded by the *Firmicutes* (low G+C Gram-positive) bacteria and their phages. The use of a sequence similarity network and gene context analyses allowed us to propose a new dataset of phage-related SSAPs, to significantly increase the amount of annotated SSAPs and to demonstrate the relationships between them. We recognized the Sak3-like proteins as a new distinct SSAP family. In result, the identified SSAPs were classified into seven distinct families, namely RecA, Gp2.5, RecT/Redβ, Erf, Rad52/22, Sak3 and Sak4, organized into three superfamilies. Here, we also suggest that bacterial phage-defense systems specifically targeting phage *ssap* genes could contribute to evolution and dissemination of the *ssap* genes among different phages and bacteria.

## Materials and Methods

### SSAPs Identification and Clustering

Sequences of SSAPs belonging to seven families: RecA (PF00154), DUF2815 (Gp2.5, PF10991), RecT/Redβ (PF03837), Erf (PF04404), Rad52/Rad22 (PF04098), DUF1071 (PF06378), and AAA_24 (Sak4, PF13479) were identified using Psi-BLAST searches (3 iterations, inclusion threshold *E*-value 0.001) against the non-redundant (nr) protein sequence database with corresponding family consensus sequences as queries. Partial protein sequences and hits from unknown organisms (e.g., Prokaryotic virus, *Siphovirus* phage, *Firmicutes* bacteria, etc.) were removed after manual inspection. In order to study detailed taxonomic distribution of identified proteins, also redundant hits were assessed. Redundant sequences present in both phage and bacterial genomes have a high chance to indicate a prophage and were separated into individual entries. The resultant set of SSAPs was clustered using CLuster ANalysis of Sequences (CLANS) to identify subfamilies of closely related SSAP sequences and elucidate the relationships between and within the SSAP families (Frickey and Lupas, 2004). CLANS runs BLAST on given sequences, all-against-all, and clusters them in 3D according to their similarity. A 2D-representation was obtained by seeding sequences randomly in the arbitrary distance space.

### Gene Context Analysis

In order to confirm the functional annotation of identified SSAP proteins [as suggested by Saha et al. (2020)], immediate genomic neighborhood (three upstream and three downstream genes) for each phage-encoded hit was retrieved from the NCBI database and manually inspected. Bacterial SSAPs were omitted because curation of such a vast dataset would be unfeasible, not to mention that many bacterial hits come from whole-genome sequencing (WGS) data resulting in a number of scattered contigs which hinders reconstruction of genomic neighborhood. Protein domains encoded by the neighboring genes were identified using pfam_scan.pl script that wraps HMMER searches against Pfam database (ver. 32).

### Identification of Abi- and P2-EC30 RT-Related Proteins

Homologs of the *L. lacti*s AbiA (AAA65072.1) and AbiK (WP_011117213.1) and *Enterobacteria* phage P2-EC30 reverse transcriptase (CAJ43154.1) proteins were retrieved using Psi-BLAST searches (five iterations, inclusion threshold *E*-value 0.05) against the non-redundant (nr) protein sequence database. Partial protein sequences were removed manually. The remaining sequences were clustered using CLANS in order to assess the distinction between highly homologous AbiA-, AbiK-, and P2-EC30-like proteins. For hits obtained after the first iteration, the gene context was inferred from the NCBI to confirm that they are functional homologs of the query proteins.

### CRISPR Spacer Analysis

Spacers corresponding to the sequences (proto-spacers) of SSAP-encoding genes were identified by BLASTn searches (at default parameters) against the CRISPRdb database^1^ (Grissa et al., 2007). Based on previous reports (Anderson et al., 2011), spacers “matches” were considered given 100% of identity along at least 20 bp. Sequences complying with less stringent thresholds (92–99% identity corresponding to one or two mismatches over the spacer length) were retained for further analyses, but regarded as degenerated. Each *ssap* proto-spacer sequence was compared for the number of matching spacers and the organism (phylum) from which they derive.

## Results and Discussion

### Construction and Analysis of the *Firmicutes* SSAP Dataset

In total, we identified more than 35,100 SSAP proteins from *Firmicutes* bacteria (bacterial subset) and their specific phages (phage subset) (Table 1). Notably, less than half of the identified phage SSAPs is annotated in the updated version of the ACLAME database comprising all known prophages and phage genomes (Leplae et al., 2010). The majority of SSAP sequences present in the newly constructed dataset (Supplementary Data Sheet 1) is encoded by chromosomal genes. However, we suggested that the bacterial subset includes unannotated prophages and phage remnant elements allocated, due to their localization, in this fraction of the SSAP dataset.

**TABLE 1:** *Firmicutes* SSAP families identified in this study.

Superfamily (Pfam Clan)	SCOP fold	Pfam ID	Family names	No. of Bacterial *ssap* sequences	No. of Phage *ssap* sequences
OB	OB-fold	PF10991	Gp2.5 Gp1.2 DUF2815	5,861	126
P-loop_NTPase	P-loop containing nucleoside triphosphate hydrolases	PF00154	RecA	nd	229
		PF13479	Sak4	6,737	339
DSRM	dsRBD-like	PF04404	Erf	5,358	216
		PF06378	Sak3 (DUF1071)	3,499	233
		PF04098	Rad52/22	440	6
		PF03837	RecT Redβ	10,517 1,375	212 80

**FIGURE 1:**
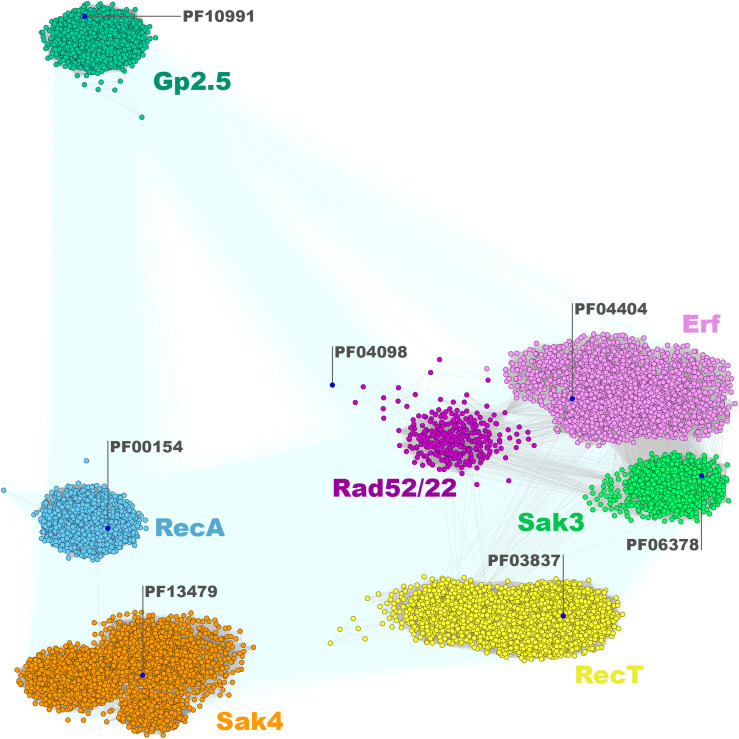
Clustering of identified full-length SSAPs encoded by *Firmicutes* bacteria and their phages. Consensus sequences for Pfam families are marked with blue dots. Connections with *P*-value lower and higher than 1E-16 are rendered in gray and light teal, respectively.

The sequences from the SSAP dataset were compared all-against-all with BLAST to find clusters of similar sequences and visualize their sequence relationships, as implemented in the CLANS (CLuster ANalysis of Sequences) program (Frickey and Lupas, 2004). This global analysis showed that SSAPs from *Firmicutes* and their phages belong to seven well-defined protein families: RecA (PF00154), Gp1.2 (DUF2815; PF10991), RecT/Redβ (PF03837), Erf (PF04404), Rad52/22 (PF04098), Sak4 (AAA_24; PF13479), and Sak3 (DUF1071; PF06378) (Figure 1). Importantly, our analysis revealed Sak3 as a distinct SSAP family, which carries representatives previously classified to Rad52/22 or Sak protein clusters (Lopes et al., 2010).

Thus, all actually identified phage SSAPs are present in our dataset. Not surprisingly, they do not display any regularity in the phyletic distribution. The SSAP families and subgroups within them are formed by proteins from unrelated phages infecting different bacterial species (Supplementary Data Sheet 1). These observations indicate an interesting particularity in the evolution of the SSAP-encoding genes as sequence similarity between phage proteins from different species and infecting diverse hosts tends to be very limited (Hendrix et al., 1999; Cresawn et al., 2011). On the other hand, similar phages infecting related hosts encode SSAPs belonging to different families. Thus, *Firmicutes* SSAPs constitute an example of an almost ubiquitous but extremely diverse and randomly distributed group of proteins.

**FIGURE 2:**
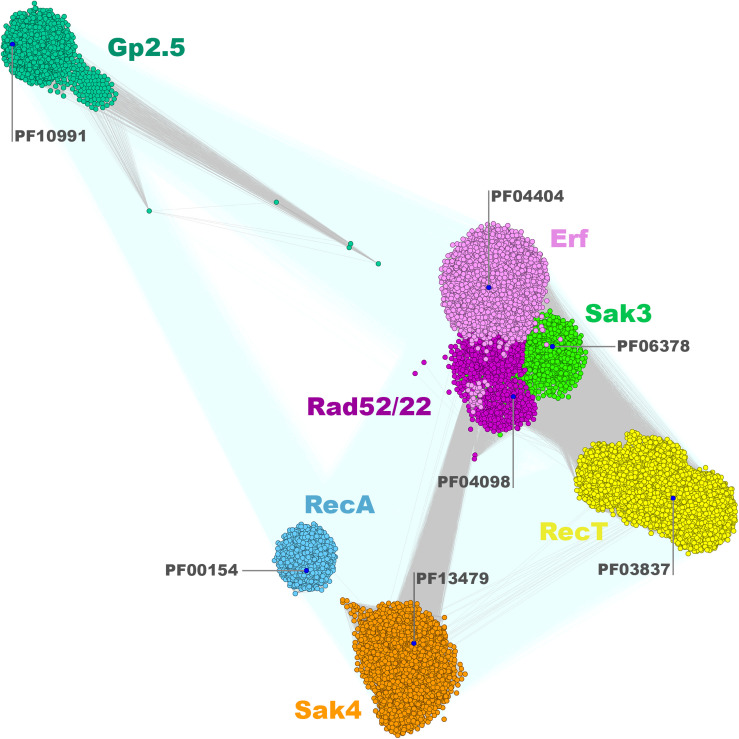
Clustering of identified full-length bacterial and phage SSAPs. Consensus sequences for Pfam families are marked with blue dots. Connections with *P*-value lower and higher than 1E-16 are rendered in gray and light teal, respectively.

The clustering of the *Firmicutes* SSAP subset matches the overall SSAP sequence similarity network calculated for all Gram-positive and Gram-negative bacterial proteins in general (Figure 2). When considering SSAPs from the Bacteria kingdom, Erf, Sak3, and Rad52/22 families are much more tightly connected, if not overlapping to some extent. In turn, those three families are more pronounced within the *Firmicutes*-only datasets. Moreover, more connections with better *P*-value emerge between Sak4, RecT, and Erf/Sak3/Rad52/22 for the Bacteria subset. Connected representatives from these distinct clusters share conserved, additional C-terminal DNA-binding domain (HTH) remotely homologous to the Redβ C-terminal domain [pdb| 6m9k (30624736)] and ParD DNA binding protein [pdb| 2an7 (17656583)]. This might be a consequence of protein domain shuffling between distinct phages that employ different SSAPs but co-infected the same host. The observed overall agreement between bacterial clustering and the *Firmicutes* subset confirms that *Firmicutes* SSAPs follow the evolutionary history of all SSAPs found in Bacteria. This allows us to assume that results obtained for the *Firmicutes* subset would be also valid for SSAPs in general.

### RecT/Redβ Superfamily

**FIGURE 3:**
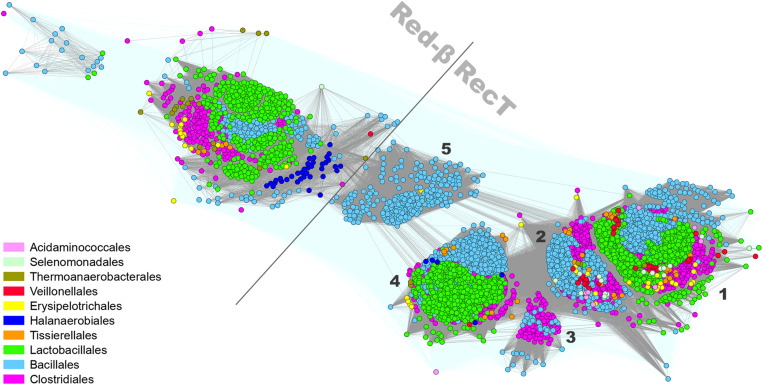
Clustering of identified full-length RecT/Redβ proteins encoded by *Firmicutes* bacteria and their phages. Connections with *P*-value lower and higher than 1E-30 are rendered in gray and light teal, respectively.

Proteins belonging to the *Firmicutes* RecT/Redβ family (Iyer et al., 2002; Lopes et al., 2010) form six well-defined subgroups: one Redβ-like subfamily of around 1,450 proteins and five RecT-like clusters altogether spanning 10,729 homologs (Figure 3). Most of the Redβ-like proteins were identified in the genomes of *Streptococcus* sp. (*Lactobacillales*), *Listeria* sp. (*Bacillales*), and *Clostridiales*. The relatively limited occurrence of Redβ-like proteins within *Firmicutes* Redβ-RecT family presents a contrast to the entirety of *Proteobacteria* where these proteins over count RecT (Supplementary Figure 1). This could suggest a relatively recent HGT of the *red* genes from Gram-negative to Gram-positive bacteria. Five subgroups of the RecT family are formed mainly by proteins encoded by: cluster 1 – *Bacillales* (*Bacillus* sp. and *Listeria* sp.), *Lactobacillales* (mainly *Enterococcus* and *Streptococcus* sp.) and *Clostridiales*, cluster 2 – *Bacillales* (mainly *Bacillus* sp.) and *Clostridiales*, cluster 3 – *Clostridiales* (*Clostridium* sp.) and *Bacillales* (*Paenibacillus* sp.), cluster 4 – *Bacillales* (mainly *Staphylococcus* sp.) and *Lactobacillales*, cluster 5 – *Bacillales* (mainly *Listeria* sp.) (Supplementary Data Sheet 2). Remarkably, cluster 5 is well separated from the other RecT clusters and localized in-between Redβ and RecT both when analyzing *Firmicutes* and *g-Proteobacteria* protein sets. Only three clusters (1, 2, and 4) contain SSAPs from virulent phages while all other members of the RecT/Redβ protein family derive from recognized prophages or are chromosomally-encoded.

In *Firmicutes*, genes coding for phage RecT/Redβ proteins are localized in proximity to genes engaged in DNA replication, e.g., phage replisome organizer with N-terminal replication initiation domain (Phage_rep_org_N; PF09681) associated with either its C-terminal, low complexity helical domain (rep_org_C, PF06926), replication initiation, and membrane attachment domain (DnaB_2, PF07261) or replication helicases (IstB_IS21-like domain). Three most common direct genomic neighbors include: the YqaJ-like (PF09588) nuclease encoding-gene, adjacent to *recT* found mostly in *Bacillales* and *Lactobacillales* phages (cluster 1 and few records in cluster 2 and 4), *duf3799*, encoding a PDDEXK-like protein (PF12684), which accompanies *recT* genes mainly in the *Lactobacillales* phage subset (cluster 1 and 4), or *duf1351* (PF07083), located next to either *red*β or *recT* genes of *Lactobacillales* phages (4 cluster). Other functions encoded in direct proximity of the phage-encoded *recT/red*β genes include SSB (PF00436, single-strand binding protein), SMC (PF02463, structural maintenance of chromosome-like protein) or further uncharacterized AAA_ATPases (Supplementary Data Sheet 1). In Gram-negative bacteria and their phages, *recT* is found adjacent to gene encoding a *recE-*like exonuclease, together forming the *recET* module involved in homologous recombination (Chang and Julin, 2001), used for recombineering, and functionally analogous to the *red*β*/exo* module of phage λ (Muyrers et al., 2000). RecE exonucleases assist RecT in catalyzing recombination by digesting DNA extremity in 5′–3′ direction (Court et al., 2002). The co-occurrence of the *recT* gene either with YqaJ-like or DUF3799 *exo* is typical for all phage RecT subgroups in *Firmicutes*, except for certain lactococcal and *Streptococcus* phages with the *duf1351* gene instead. The combination *recT-lactamase B2* is characteristic specifically for *Bacillales* prophages (of *Paenibacillus* sp., *Bacillus* sp., *Staphylococcus* sp. hosts) that lack a recognizable *exo* in the proximity of the *ssap* genes. This finding together with the confirmed role of phages in dissemination of drug resistance genes, such as lactamase genes, *via* the HGT (Muniesa et al., 2004) suggests the spread of the whole *recT-lactamase B2* by this manner. The topological arrangement within the Redβ subgroup shows the *ssap-duf135*1 gene order to be the most common. Analysis of the present *Firmicutes* SSAP dataset did not reveal any member of the EHAP1-like protein family previously identified in *E. coli* and *Salmonella* as a distinct subgroup of the RecT/Redβ superfamily (Iyer et al., 2002). Given all of the identified SSAP clusters examined throughout this study (see below), the proteins from RecT/Redβ family can be detected in over 100 genera and seem to be the most widely distributed among *Firmicutes* taxons.

### Rad52/22-Erf-Sak3 Superfamily

Both the Erf-like and Rad52/22 proteins have been recognized as members of the second SSAP superfamily, Rad52/22-Erf (Lopes et al., 2010). Erf proteins are abundant among the identified SSAPs (216 viral subset members; Table 1). Based on CLANS comparison, the Erf protein family is also one of the most diffused clades of the Rad52/22-Erf superfamily (Figure 1). The greater fraction of these proteins is encoded by temperate phages, prophages or phage remnants residing in *Firmicutes* bacterial genomes of *Lactobacillales* (mainly *Lactobacillus* spp. and *Enterococcus* spp.), *Bacillales* (mainly *Staphylococcus* spp.) and *Clostridiales*. Seventy-seven proteins were found to be encoded by virulent phages, most of which infect *Lactococcus* species (Supplementary Data Sheet 1). This revises the previous view (Lopes et al., 2010) that Erf-like proteins are strictly specific to temperate phages.

Examination of gene neighborhoods of the phage subset of *Firmicutes erfs* revealed the most frequent association (almost 80%) with *ssb* genes. Other possible downstream genes that may assist *erf* vary and include *rep*, *hnh endo*, *phg_2220_C* (Supplementary Data Sheet 1). In the majority of cases, the *erf-ssb* module is neighbored upstream by a gene encoding: (i) the homolog of phage Mu DNA binding protein Gam (Akroyd and Symonds, 1986), (ii) DUF2483- or (iii) DUF1351-type protein, or (iv) the homolog of *S. thermophilu*s phage øSfi21 *orf157* product (*sipho_gp157*) (Foley et al., 1998). The common localization of these genes in respect to the *erf-ssb* module implies their analogous function. The *duf2483-erf* module seems to be restricted to *Staphylococcus* phages, while *duf1351-erf* to *Lactobacillus* phages.

In contrast to Erfs, which are encoded in the genomes of various *Firmicutes* bacterial hosts, temperate and lytic phages, Rad52/22 sequences were found only in a few genera. Rad52/22 homologs were identified within temperate phages and prophage sequences of bacterial hosts from *Lactobacillales* (*Lactococcus lactis*), *Bacillales* (mainly *Bacillus* spp.), and *Clostridiales* (mainly *Clostridium spp*.) order. The only virulent phages encoding a Rad52/22-like protein are members of the lactococcal phage P335 group, which carry a non-functional, lysogeny module (Labrie and Moineau, 2002; Mahony et al., 2013). Firm clustering of Rad52/22 members indicates strong sequence conservation within this protein family and suggests their close phylogenetic relationships. Remarkably, the *Firmicutes* Rad52/22 cluster is shifted away from the consensus sequence for this Pfam family (Figure 1), suggesting sequence divergence of this subgroup from proteins found in other, non-Gram positive bacteria.

We found that the genetic context of the phage subset of *rad52/22* genes is similar to the organization of the *erf* recombination modules (Supplementary Data Sheet 1), which are adjacent to *ssb* gene (*rad52/22-ssb* module), followed directly downstream by *rep* functions. The third distinct SSAP clan (Figure 1) identified by our CLANS analysis regroups proteins related to Sak3 SSAP (*gporf35*) from the virulent lactococcal phage p2 (Bouchard and Moineau, 2004; Scaltriti et al., 2011). This protein was classified earlier as a member of the Rad52/22 SSAP family on the basis of the prediction of their structural folds (Lopes et al., 2010). However, we have established that Sak3-like proteins (formally a DUF1071 family of proteins of unknown function) form an individual distinct SSAP clan. Majority of them constitute the bacterial SSAP subset and are encoded in large part in *Bacillales* (mainly *Staphylococcus* spp.) genomes (Supplementary Data Sheet 1 and Figure 1). In turn, 90% of the DUF1071 sequences representing the phage subset are associated with the genomes of small isometric-headed, lytic lactococcal phages. In general, proteins from the Sak3/DUF1071 phage subset seem to be characteristic for lytic *Firmicutes* phages and, besides them, were found in phages of high G+C Gram-positive bacteria (*Mycobacteria –* 88 phages and *Actinobacteria* – 1 phage) and in few vibrio- and cyanophages infecting Gram-negative hosts (Supplementary Data Sheet 1). The less frequent occurrence (∼10-fold) of DUF1071-encoding genes in Gram-negative bacteria and phages might indicate a recent expansion of these proteins uprooting from Gram-positive organisms.

We found that like *erf* and *rad52*/22, *sak3*/*duf1071* sequences are often associated with the same genes. The *duf1071-ssb* organization is prevalent, except for lactobacilli phages which possess the *duf1071-phg_2220* module (Supplementary Data Sheet 1). In the genomes of virulent lactococcal phages, *duf1071* is associated with a gene encoding a particular type of SSB protein (Szczepankowska et al., 2011). In staphylococcal phage genomes, the *duf1071-ssb* modules are typically followed by genes encoding proteins with the HNHc_6 domain, followed by *rep* genes; thus, forming a *duf1071-ssb-hnhc_6-rep* module. We observed a similar organization in some staphylococcal phages encoding the Erf protein. Yet, in contrast to staphylococcal *erf* modules, *duf1071* is always preceded by *duf2483*. In turn, in lactococcal phages, the *duf1071-ssb* module is most often neighbored upstream by genes encoding homing nucleases (NUMOD1/HNH_3/NUMOD4). Interestingly, the gene context typical for Gram-positive bacteria is not conserved in Gram-negative species where the *duf1071* gene was detected. These genes seem not to be located in the vicinity of other prophage genes, but rather within the transposon-like regions (data not shown).

Sak3/DUF1071 proteins exhibit relevant sequence similarity with both Erf and Rad52/22 proteins, which is supported and elegantly visualized by our CLANS analysis. The finding that *sak3/duf107*1 genes are often positioned in the same genetic context as *erf* and *rad52/22* genes emphasizes the orthology between these three types of SSAPs and implies coinciding roles of all three proteins in recombination processes. It probably provides an explanation for frequent HGT detected for members of this superfamily.

### Sak4 and RecA Families

Genes encoding Sak4-like proteins, initially identified in the genomes of lactococcal phages phi31 (*orf245*) and TPW22 (Bouchard and Moineau, 2004), represent a novel type of SSAPs not related to Rad52/22-Erf-SAK3-like superfamily. Instead, Sak4 proteins show much higher similarity with phage-encoded RecA recombinases and retain signatures characteristic for the RecA superfamily, e.g., catalytic glutamate in the P-loop domain and an arginine-finger from a C-terminal β-hairpin (Leipe et al., 2000). However, they were recognized as a distinct group from the classical RecA proteins based on the presence of a minimal version of the ATPase domain (de Souza et al., 2010). In our database, Sak4 family pools more than 330 members encoded both by lytic and temperate *Firmicutes*-infecting phages and around 6,700 by *Firmicutes* bacteria, mainly *Bacillales* (*Staphylococcus* spp.) and *Lactobacillales* (*Lactobacillus* spp.) (Figure 1 and Supplementary Data Sheet 1). We found that the phage *sak4* genes are most commonly localized upstream of genes encoding SSB proteins, including DUF669—a distant homolog of SSB of phages N4, Lc-Nu, and PhiAT3 (Hutinet et al., 2018) and DNA helicases (resIII/helicase_C domain). Similar to *erf*, the upstream gene neighbors of *sak4* include: phage Mu *gam-*like genes, *S. thermophilu*s phage øSfi21 *orf157*-like genes and *duf2483* or *duf1351*. The *duf2483-sak4* module seems to be restricted to *Staphylococcus* phages, while *duf1351-sak4* to *Streptococcus* phages. In several cases, *sak4* of streptococcal phages is accompanied by the *sbcC* exonuclease gene.

As for the canonical RecA, we found that the phage proteins are encoded in great majority by large virulent broad-host range *Herelleviridae* phages specific for *Bacillales* (mainly *Staphylococcus* spp., but also *Bacillus* spp. and *Listeria* spp.), recognized as SPO1-like viruses (Klumpp et al., 2010; El-Arabi et al., 2013; Barylski et al., 2014). These phages retain common morphological properties, their genomes consist of a terminally redundant, non-permuted dsDNA molecule of 127–200 kb in size and share considerable amino acid homology. Yet, it seems that the RecA recombinase is not a primary feature of large SPO1-related phages as some other large virulent phages infecting *Bacillus* sp. and *Staphylococcus* sp. deposited in GenBank do not encode for this protein. We determined that the genetic context of phage *recA* genes is characterized by the proximity of a putative *ssb* and a gene encoding phage specific RNA polymerase sigma factors. Other genes that can be found in close vicinity of *recA* vary and include *orfs* with mostly unknown functions, but also phage DNA *polA* and ligase genes (Supplementary Data Sheet 1).

### Gp2.5 Family

Based on our analyses, we determined that protein members homologous to phage T7 Gp2.5 protein form a very compact clan distinct from other SSAPs (Figure 1). Within *Firmicutes* bacteria, Gp2.5-like SSAPs are mostly present in *Bacillales* (mainly *Staphylococcus* spp.), *Clostridiales* (mainly *Clostridium* spp.) and, to a lesser extent, in *Lactobacillales* (mainly *Streptococcus* spp.). In the phage subset, we detected the highest number of Gp2.5 homologs in the genomes of streptococcal and staphylococcal phages (Supplementary Data Sheet 1). In phages, *gp2.5* is often located in proximity to DNA *polA* and *duf2800* (PD-(D/E)-XK nuclease-like gene) (Steczkiewicz et al., 2012), altogether forming the *duf2800-gp2.5-polA* module. Interestingly, an identical gene context was also detected in the majority of phages of Gram-negative bacteria (data not shown). The distinctive feature of the *gp2.5* genetic neighborhood is the absence of *ssb* genes. High level of sequence similarity, restricted phyletic distribution and conserved genetic context, altogether indicate that the Gp2.5 family has emerged recently or displays low/stable evolution rates.

### Diversity of Phage SSAPs and Bacterial Phage Defense Mechanisms

The present study demonstrates that *Firmicutes* bacteria and their phages possess diverse SSAPs which are distributed irregularly across different phyla suggesting frequent horizontal transfer of the corresponding genes. The high diversity of SSAPs is in stark contrast to the ubiquitous and highly conserved RecA recombinase which is one of the most slowly evolving proteins (Eisen, 1995) and shows a low rate of horizontal transfer (Rocha et al., 2005). High rates of molecular evolution have been found for genes, which are constantly subjected to strong selective pressure, especially genes associated with infection and resistance to infection (Buckling and Rainey, 2002; Labrie and Moineau, 2007; Paterson et al., 2010). Thus, one can suggest that phage *ssap* genes, which are crucial for phage multiplication, represent a target for commonly distributed bacterial resistance mechanisms. Consequently, this could stimulate the generation of the high variety and sequence diversity among *ssap* genes.

In order to counterfeit phage infection, bacteria developed multiple defense strategies, including CRISPR/Cas and Abortive infection (Abi) systems. CRISPR/Cas systems efficiently prevent phage multiplication by using spacer sequences homologous to regions in the phage genomes. A specific spacer guides the Cas nuclease to cleave the complementary phage nucleic acid. The CRISPR spacers are apt to change in time so that the system loses its specificity toward a particular phage sequence (Sorek et al., 2008). Thus, one could not expect to detect a significant number of spacers identical to the *ssap* sequences if they are not regularly re-acquired.

**FIGURE 4:**
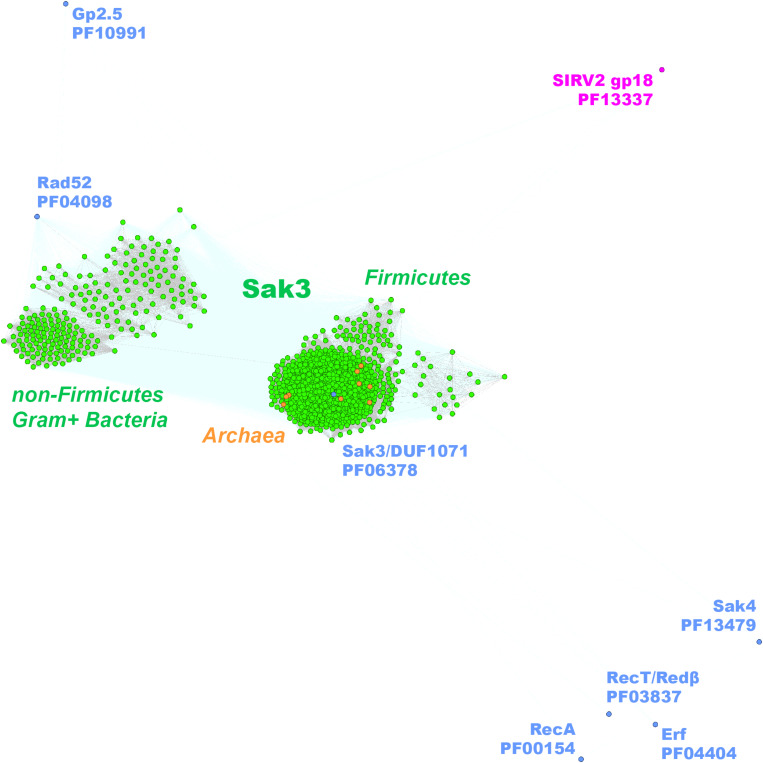
Clustering of the identified full-length Gram+ bacteria (in green) and Archaea (in orange) Sak3 (DUF1071-like) proteins. Consensus sequences for the other SSAP families are marked with blue dots. SSAP-like protein of *S. islandicus* virus SIRV2 (ORF436) is indicated in pink.

To investigate whether *ssap* genes could be targeted by CRISPR/Cas, we compared 1,388 sequences from the *Firmicutes* phage SSAP dataset to spacer sequences from the CRISPRdb database (Grissa et al., 2007). Fifty-one *ssaps* sequences from the phage Gp2.5, Sak4, Erf, RecT/Redβ, and RecA SSAP subgroups matched sequences of spacers from Bacteria and Archaea (Supplementary Data Sheet 3). Most of the spacers derived from *Firmicutes* bacteria and matched sequences from phages that infect them (Table 2). Remarkably, no spacers from *Firmicutes* bacteria matching the *duf1071*-like or *rad52/22 ssap* genes could be detected. This was quite surprising, especially for *duf1071*, which is a prevalent *ssap* type among lytic phages of *Lactococcus lactis* species. CRISPR spacers may degenerate, but still can be regarded as a sign of past infections (Andersson and Banfield, 2008). Thus, we also searched for sequence “matches” exhibiting lower identity (up to two mismatches). After loosening the search criteria, we established that *duf1071* genes from lactococcal phages were most frequently detected as matches to spacer sequences, whereas the spacers themselves derived from archaeal genomes (Supplementary Data Sheet 3). This finding opens the discussion of the possible evolutionary link between lactococcal phages and Archaea. Our previous study provided evidence that SSB proteins encoded by lytic lactococcal phages might have archaeal origin (Szczepankowska et al., 2011). Genes encoding SSAP and SSB proteins define the recombination modules in many phage genomes (Szczepańska et al., 2007; Szczepankowska et al., 2011; Neamah et al., 2017). Also, the only functionally characterized SSAP, ORF436 (gp18), of an archaeal phage *Sulfolobus islandicus* SIRV2 is encoded directly downstream of an *ssb* gene (Guo et al., 2015). Despite the presence of different domains within SIRV2_ORF436 (Lon_2 protease-like domain, PF13337) and DUF1071-like proteins (dsRBD-like domain, PF06378), the conserved position of *ssap-ssb* gene module in both, Bacteria and Archaea suggests similar function. Our idea on the possible archaeal origin of now deteriorated, matching spacer sequences necessitates further studies but seems to be reinforced by identification of *duf1071*-like genes in several archaeal genomes (Supplementary Data Sheet 1), which all cluster tightly with the *Firmicutes* Sak3/DUF1071 family (Figure 4).

**TABLE 2:** CRISPR spacers that match to the *Firmicutes ssap* genes.

Spacer	*ssap* match	Spacer match (nt)	Source organism
**Sak4-like**			
NC_007432_1_6	*Streptococcus* phage Javan17 *Streptococcus* phage Javan31 *Streptococcus* phage Javan46	30	*S. agalactiae* A909
NC_007432_1_6	*Streptococcus* phage Javan47	30	*S. agalactiae* A909
NC_008022_1_1	*Streptococcus* phage 6180.1 *Streptococcus* phage Javan474 *Streptococcus* phage Javan484 *Streptococcus* phage Javan516	30	*S. pyogenes* MGAS10270
NC_008022_1_1	*Streptococcus* phage Javan7 *Streptococcus* phage LF4	30	*S. pyogenes* MGAS10270
NC_008022_1_1	*Streptococcus* phage JX01	30	*S. pyogenes* MGAS10270
NC_008022_1_1	*Streptococcus* phage LF1	30	*S. pyogenes* MGAS10270
NC_017568_1_15	*Staphylococcus* phage phiSP15-1	28	*S. pseudintermedius* ED99
NZ_CP007573_2_32	*Streptococcus* phage Javan59	30	*S. anginosus* GCF_000831165
NZ_CP007573_2_32	*Streptococcus* phage Javan68	30	*S. anginosus* GCF_000831165
NZ_CP010874_2_3 NZ_CP010875_2_2 NZ_CP012419_2_2 NZ_CP016391_2_6	*Streptococcus* phage Javan17 *Streptococcus* phage Javan31 *Streptococcus* phage Javan46	30	*S. agalactiae* GCF_001683515
NZ_CP010874_2_3 NZ_CP010875_2_2 NZ_CP012419_2_2 NZ_CP016391_2_6	*Streptococcus* phage Javan253	20	*S. agalactiae* GCF_001275545
NZ_CP010874_2_3 NZ_CP010875_2_2 NZ_CP012419_2_2 NZ_CP016391_2_6	*Streptococcus* phage Javan460 *Streptococcus* phage Javan488 *Streptococcus* phage Javan506 *Streptococcus* phage 370.1	30	*S. agalactiae* GCF_001592425
NZ_CP010874_2_3 NZ_CP010875_2_2 NZ_CP012419_2_2 NZ_CP016391_2_6	*Streptococcus* phage Javan47	30	*S. agalactiae* GCF_001683515
NZ_CP019794_2_3 NZ_CP020327_9_3	*Paenibacillus* phage LincolnB	34	*P. larvae* subsp. *pulvifaciens* GCF_002007765
NZ_CP019794_2_3 NZ_CP020327_9_3	*Paenibacillus* phage Wanderer	34	*P. larvae* subsp. *pulvifaciens* GCF_002007765
**Erf-like**			
NC_011134_4_10 NC_011134_4_13	*Streptococcus* phage Javan179	30	*S. equi* subsp. *zooepidemicus* MGCS10565
NC_017174_12_24	*Clostridium* phage phiCDHM19	37	*C. difficile* M120
NC_017576_2_18	*Streptococcus* phage Javan226	30	*S. gallolyticus* subsp. *gallolyticus* ATCC 43143
NZ_CP019687_3_4	*Paenibacillus* phage Harrison *Paenibacillus* phage Paisley	36	*P. larvae* subsp. *larvae* GCF_002003265
**Gp2.5-like**			
NC_014377_4_7	*Clostridium* phage phiCT453A	22	*T oceani* DSM 16646
NC_014377_4_7	*Erysipelothrix* phage phi1605	22	*T. oceani* DSM 16646
**RecT/Redβ-like**			
NC_004368_1_9	*Streptococcus* phage Javan384	26	*S. agalactiae* NEM316
NC_012891_2_9	*Streptococcus* phage 370.2 *Streptococcus* phage Javan459 *Streptococcus* phage Javan489 *Streptococcus* phage Javan525	25	*S. dysgalactiae* subsp. *equisimilis* GGS_124
NC_012891_2_9	*Streptococcus* phage Javan451	25	*S. dysgalactiae* subsp. *equisimilis* GGS_124
NC_014206_6_10	*Bacillus* phage vB_BspS_SplendidRed	20	*Geobacillus* sp. C56-T3
NC_017174_6_5	*Clostridium* phage CDKM9	37	*C. difficile* M120
NC_018712_3_12	*Streptococcus* phage Javan141	36	*S. dysgalactiae* subsp. *equisimilis* RE378
NC_021900_2_6	*Streptococcus* phage Javan202	21	*S. lutetiensis* 033
NC_021900_2_6	*Streptococcus* phage Javan215	25	*S. lutetiensis* 033
NC_021900_2_6	*Streptococcus* phage Javan263	20	*S. lutetiensis* 033
NZ_CP015408_1_1	*Lactobacillus* phage LR1	23	*Lb. reuteri* GCF_001688685
NZ_CP017267_2_7	*Brochothrix* phage NF5	26	*V. teuberi* GCF_001870205
NZ_CP017267_2_7	*Streptococcus* phage Javan384	23	*V. teuberi* GCF_001870205
**RecA**			
NZ_CP009687_2_45	*Staphylococcus* phage phiSA_BS1	22	*C. aceticum* GCF_001042715
NZ_CP009687_2_45	*Staphylococcus* phage phiSA_BS2	22	*C. aceticum* GCF_001042715

*Spacers are named by “genome. locus. spacer-number” (counted from the leader end). SSAP family and origin (*ssap* match) name are given along with the length of the spacer match and source organism. Only spacers derived from bacteria that match *ssap* genes with 100% identity are listed.*

Overall, our analysis shows that spacer sequences matching *ssap* genes represent a non-negligible quantity, especially taking into account that a bulk of almost 9.000 CRISPR spacers from the CRISPRdb database does not match any known sequences (Grissa et al., 2007; Shmakov et al., 2017). In accordance with previous communications, this may reflect (repeated) acquisition of specific spacers based on their effectiveness (Paez-Espino et al., 2013).

Given that only one CRISPR/Cas system has been identified in *Lactococcus lactis* (Millen et al., 2012), we asked whether other phage resistance mechanisms could specifically target the *ssap* genes of *Firmicutes* bacteria. Several SSAPs encoded by lactococcal phages, hereby classified as members of Erf, Rad52/22, Sak3/DUF1071, and Sak4 protein families, were already experimentally confirmed as targets of the phage abortive infection mechanism, AbiK (Emond et al., 1997; Boucher et al., 2000, 2001; Bouchard and Moineau, 2004; Fortier et al., 2005; Scaltriti et al., 2010, 2011). One of them, the Sak4 protein encoded by phage phi31, has also been recognized as a target for another abortive infection mechanism—AbiA (Garvey et al., 1995; Dinsmore and Klaenhammer, 1997).

Both Abi mechanisms are active against lactococcal phages from three most abundant and genetically unrelated groups (936-like small isometric-headed phages, c2-like prolate-headed phages, and mostly temperate P335-like phages) (Deveau et al., 2006; Mahony et al., 2013). The AbiA system has also been demonstrated to block multiplication of six tested *Streptococcus thermophilus* phages (Tangney and Fitzgerald, 2002), of which one (phage 7202) was annotated to possess the *erf* gene (NC_002185). Up to now, only four proteins highly homologous to the *L. lactis* AbiK have been annotated as putative abortive infection agents (Wang et al., 2011). However, both lactococcal *abiK* and *abiA* are plasmid-encoded genes which might facilitate their wide distribution among related Gram-positive bacteria, such as the aforementioned *S. thermophilus* species. Here, we identified *abiK* and *abiA* homologous genes on over fourty and five plasmids, respectively, found in several different *Firmicutes* species (Supplementary Data Sheet 4). Yet, the majority of *abiK* and *abiA* genes are not plasmid-encoded but are rather located on chromosomes or come from WGS data (Supplementary Data Sheet 4). Phage defense systems (e.g., restriction-modification, toxin-antitoxin modules, and CRISPR elements) tend to be clustered into genomic islands (Makarova et al., 2011) known to spread *via* horizontal transfer events (Juhas et al., 2009). By gene context analyses we revealed that *abiK-* and *abiA-*like genes are associated with diverse genes involved in phage resistance, which is suggestive for their HGT-mediated acquisition (Supplementary Data Sheet 4).

**FIGURE 5:**
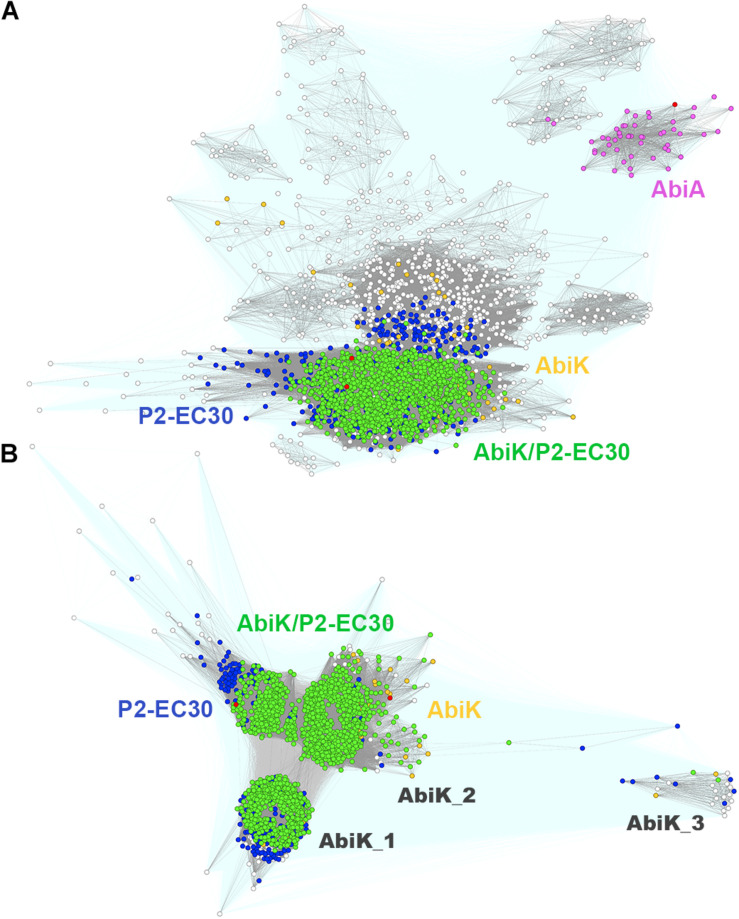
Clustering of full-length *Firmicutes* AbiK, AbiA, and P2-EC30 RT proteins **(A)**. Query sequences for AbiK, AbiA, and P2-EC30 are marked with red dots. Connections with *P*-value lower and higher than 1E-16 are rendered in gray and light teal, respectively. Colored dots denote protein sequences identified in the 1st iteration of the Psi-BLAST search. Sub-clustering of full-length *Firmicutes* AbiK and P2-EC30 RT proteins **(B)**. Query sequences for AbiK and P2-EC30 are marked with red dots. Connections with *P*-value lower and higher than 1E-36 are rendered in gray and light teal, respectively. Colored dots denote proteins identified in the 1st iteration of the Psi-BLAST with P2-EC30 RT (blue), AbiK (yellow) and either P2-EC30 RT or AbiK (green) as a query. White dots indicate proteins not detected in the 1st iteration.

Both, AbiK and AbiA exhibit high sequence similarity (23% identity and 44% of similarity) and contain the RVT_1 reverse transcriptase domain (PF00078), which has been shown to interfere with phage DNA replication (Boucher et al., 2000; Wang et al., 2011). Interestingly, the classical *L. lactis* AbiK shares homology (32% sequence identity) with yet another reverse transcriptase encoded by the *E. coli* P2-EC30 prophage (Odegrip et al., 2006). Both protein subfamilies overlap significantly, but remain clearly isolated from AbiA (Figure 5A). More detailed sequence clustering allowed to separate AbiK from P2-EC30 and reveal the presence of several AbiK subclusters consisting of proteins with probably slightly different anti-phage specificities (Figure 5B). Each of these AbiK subgroups is shown to have different phylogenetic distribution (Supplementary Data Sheet 4). The canonical AbiK proteins are present mainly in staphylococci. The biggest newly identified clusters of AbiK-like proteins include AbiK_1, mainly from *Bacillales, Clostridia*, and *Lactobacillales*, AbiK_3 proteins most of which derive from *Lactobacillus* species and AbiK_2 proteins (mainly *Bacillales* and *Clostridia*) closely related to the canonical AbiK-like group. Interestingly, despite the initial identification of *abiK* and *abiA* genes as phage resistance mechanisms active in *L. lactis*, we found that AbiA, AbiK or P2-EC30-like systems are under-represented in multiple *Lactococcus* sp. hosts. This observation may support the earlier notion that DUF1071-encoding genes, prevalent in lytic lactococcal phages, have emerged recently within *Firmicutes* and that Abi phage resistance mechanisms for these SSAPs are less frequently encountered in their bacterial hosts.

The wide distribution of the Abi systems and SSAPs across *Firmicutes* phylum implies efficient HGT of both. We suggest that the spread of AbiK- and AbiA-like protein clusters among diverse *Firmicutes* bacteria is a factor affecting the diversity and irregular dissemination of their potential phage targets, namely the members of Erf, Rad52/22, Sak3/DUF1071 and Sak4 SSAP families. Such notion is in agreement with the well-documented ability of the different phage resistance mechanisms to contribute to the evolution of lactococcal phages (Bouchard and Moineau, 2000, 2004; Durmaz and Klaenhammer, 2000; Odegrip et al., 2006; Labrie and Moineau, 2007).

We speculate that, in general, the diverging evolution of phage recombinases could be a consequence of phage-bacteria arms race, linked to the evolution of phage resistance mechanisms targeting the *ssap* genes. Certainly, experiments are required to verify this hypothesis. Phages could evade Abi- and/or CRISPR-mediated defense systems through single mutations within the target gene or *via* HGT, which may provide an alternative way to escape phage defense mechanisms directed against such genes.

## Conclusion

We significantly broadened and manually inspected the dataset of SSAP proteins encoded by *Firmicutes* and their phages. By applying detailed sequence clustering, we have defined numerous known and newly identified SSAPs that have not been annotated as such.

Based on thorough gene context analyses, we established signature functions that accompany the phage-encoded *ssap* genes. This resulted in a high quality dataset that can be further used for rational selection of individual *ssap* genes or recombination modules for application in recombineering techniques.

Results of our analyses show that phyletic distribution of genes encoding specific SSAP types is not phage- or bacteria-related. We suggest that the high genetic variability and irregular dissemination of phage SSAP-encoding genes occurs *via* HGT as a consequence of the co-evolutionary arms race between bacteria and phages. Bacterial-phage defense mechanisms, either highly specialized (Abi systems) or adaptive (CRISPR/Cas systems) that target SSAP functions can make a significant contribution to the diversity and evolution of the corresponding (*ssap*) genes. On the other hand, the presence of defense mechanisms directed against *ssaps* may interfere with viability and/or stability of strains carrying phage recombination genes and should be taken in consideration when choosing the bacteria-*ssap* pair used for recombineering.

## Data Availability Statement

The original contributions presented in the study are included in the article/Supplementary Material, further inquiries can be directed to the corresponding author/s.

## Author Contributions

KS collected SSAP sequences, performed bioinformatics analyses, prepared the figures, and wrote sections of the manuscript. EP designed the experiments and performed initial database analyses. AS manually analyzed all data, performed Abi and CRISPR spacer analyses, prepared the tables, and the main text of the manuscript. EB was responsible for the experimental concept and drafted the main text of the manuscript. KS retrieved all data from public databases and prepared Supplementary Figure 1. AS prepared the excel spread sheets and performed manual inspection of all data contained in the files. All authors have read and approved the manuscript.

## Conflict of Interest

The authors declare that the research was conducted in the absence of any commercial or financial relationships that could be construed as a potential conflict of interest.

## Footnotes

1 https://crispr.i2bc.paris-saclay.fr/
