# Supplementary material for: Expanding Diversity of Firmicutes Single-Strand Annealing Proteins: A Putative Role of Bacteriophage-Host Arms Race
Source: Front Microbiol. 2021 Apr 20;12:644622. doi: 10.3389/fmicb.2021.644622 (PMC8093625; doi:10.3389/fmicb.2021.644622)
Supplement: Supplementary file 5 [file Presentation_1.pptx]

## Slide 1
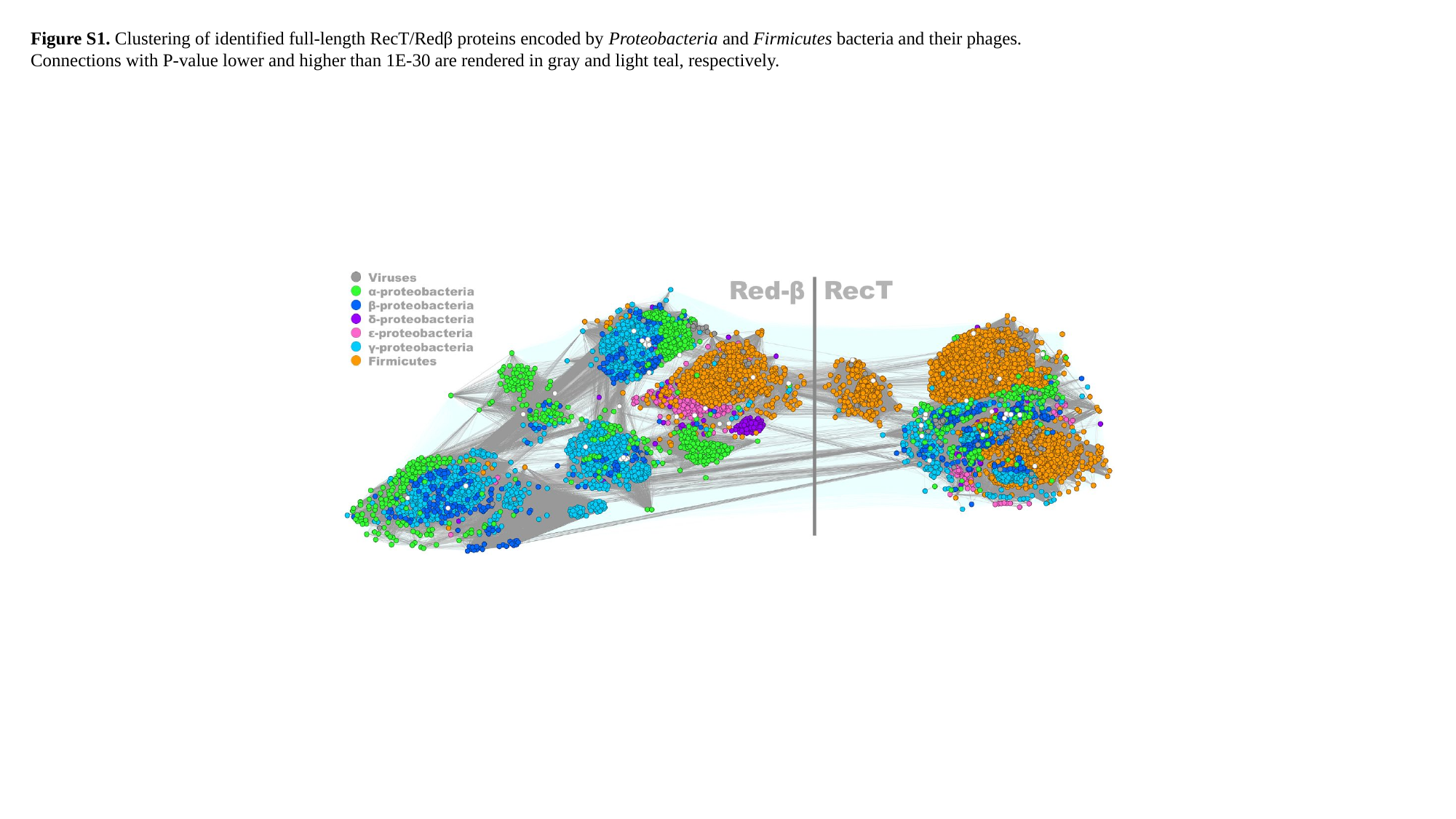

Figure S1. Clustering of identified full-length RecT/Redβ proteins encoded by Proteobacteria and Firmicutes bacteria and their phages.
Connections with P-value lower and higher than 1E-30 are rendered in gray and light teal, respectively.

## Slide 2
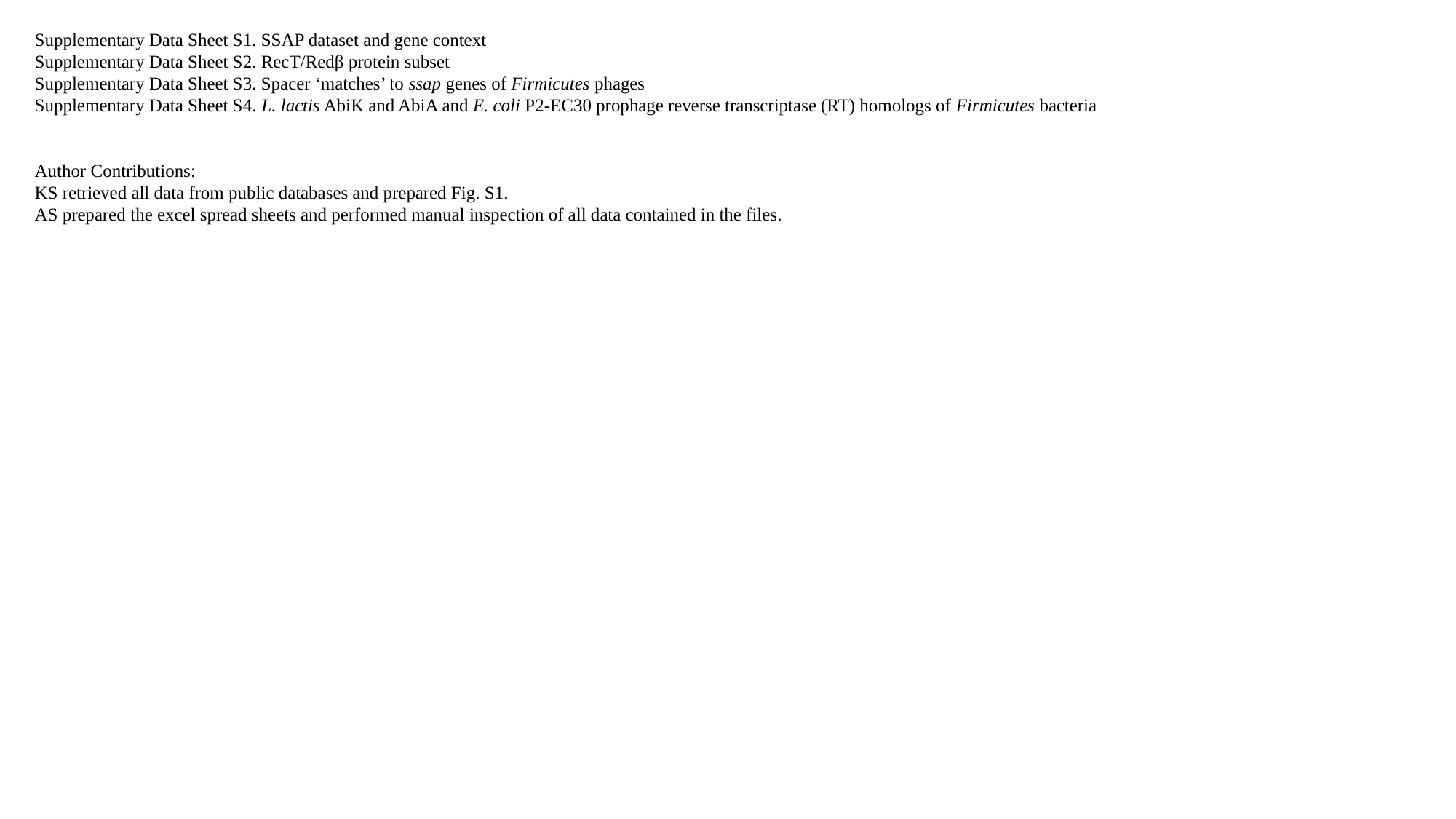

Supplementary Data Sheet S1. SSAP dataset and gene context
Supplementary Data Sheet S2. RecT/Redβ protein subset
Supplementary Data Sheet S3. Spacer ‘matches’ to ssap genes of Firmicutes phages
Supplementary Data Sheet S4. L. lactis AbiK and AbiA and E. coli P2-EC30 prophage reverse transcriptase (RT) homologs of Firmicutes bacteria
Author Contributions:
KS retrieved all data from public databases and prepared Fig. S1.
AS prepared the excel spread sheets and performed manual inspection of all data contained in the files.
